# Longitudinal diffusion tensor imaging in amyotrophic lateral sclerosis

**DOI:** 10.1186/1471-2202-13-141

**Published:** 2012-11-08

**Authors:** Carsten Keil, Tino Prell, Thomas Peschel, Viktor Hartung, Reinhard Dengler, Julian Grosskreutz

**Affiliations:** 1 Department of Neurology, Jena University Hospital, Erlanger Allee 101, Jena, 07747, Germany; 2 Department of Psychiatry and Psychotherapy, Medical School Hannover, Carl-Neuberg-Strasse 1, Hannover, 30625, Germany; 3 Department of Neurology and Clinical Neurophysiology, Medical School Hannover, Carl-Neuberg-Str.1, Hannover, 30625, Germany

**Keywords:** Cerebellum, Amyotrophic lateral sclerosis, Diffusion tensor imaging, Follow-up

## Abstract

**Background:**

Amyotrophic lateral sclerosis (ALS) is a fatal neurodegenerative disorder, caused by progressive loss of motor neurons. Changes are widespread in the subcortical white matter in ALS. Diffusion tensor imaging (DTI) detects pathological changes in white matter fibres in vivo, based on alterations in the degree (diffusivity, ADC) and directedness (fractional anisotropy, FA) of proton movement.

**Methods:**

24 patients with ALS and 24 age-matched controls received 1.5T DTI. FA and ADC were analyzed using statistical parametric mapping. In 15 of the 24 ALS patients, a second DTI was obtained after 6 months.

**Results:**

Decreased FA in the corticospinal tract (CST) and frontal areas confirm existing results. With a direct comparison of baseline and follow-up dataset, the progression of upper motor neuron degeneration, reflected in FA decrease, could be captured along the CST and in frontal areas. The involvement of cerebellum in the pathology of ALS, as suspected from functional MRI studies, could be confirmed by a reduced FA (culmen, declive). These structural changes correlated well with disease duration, ALSFRS-R, and physical and executive functions.

**Conclusion:**

DTI detects changes that are regarded as prominent features of ALS and thus, shows promise in its function as a biomarker. Using the technique herein, we could demonstrate DTI changes at follow-up which correlated well with clinical progression.

## Background

Amyotrophic lateral sclerosis (ALS) is a neurodegenerative disorder characterized by progressive failure of upper and lower motor neurons. In the ALS brain, degeneration of fibres is widespread in the subcortical white matter with surrounding astrogliosis. Degeneration commonly extends into the corticospinal tract (CST), the central part of the corpus callosum and in frontal areas as well as in long projection fibres as seen in post-mortem tissue [1,2]. Previous studies have demonstrated white matter changes in ALS using diffusion tensor imaging (DTI), a technique that detects alterations in both degree (diffusivity, ADC) and directedness (fractional anisotropy, FA) of proton movement reflecting changes in microstructural tissue and fibre organisation [3]. In ALS, DTI revealed a variable, but widespread pattern of white matter abnormalities which were most prominent along the CST and the subcentral white matter, and which extended into frontal regions [4-9].

Symptoms indicating involvment of lower motor neurons such as atrophic paresis may sometimes obscure signs of upper motor neuron failure leading to a delay in diagnosis and treatmentof ALS. Therefore, a biomarker for diagnostic, prognostic, and monitoring purposes is needed. Here, DTI shows promise as a potential biomarker, however, longitudinal studies are required to further clarify the time course and distribution of changesdetected by this technique. By customizing scripting in the Statistical Parametric Mapping software (SPM, http://www.fil.ion.ucl.ac.uk/spm) we aimed to analyze DTI datasets from 24 ALS patients, without having to make a priori assumptions about localization of pathology. To obtain longitudinal data on progress of microstructural changes in ALS, a second examination was undertaken in a subset of 15 patients at 6 months.

## Methods

### Subjects

We studied DTI image sets of 24 patients (T0) with sporadic ALS and of 24 healthy age-matched controls. In a subset of 15 of the 24 ALS patients (Follow-up), a second examination was performed 6 months later. In the 24 ALS patients, the mean (± S.D.) duration since onset of dysarthria or weakness (termed disease duration) at MRI was 25.6 (± 27,8) months, the mean age was 62.4 ± 10.5 (range 32.7–75.9) years, and the mean revised ALS functional rating scale (ALSFRS-R) was 36.3 ±9 points. The female to male ratio was 1:1. 9 patients had a bulbar onset and 15 patients had, limb onset of weakness. All patients were being treated with riluzole and none were taking psychoactive drugs. Significant frontal or cognitive dysfunctions were ruled out by means of the Mini Mental State Examination (MMSE) and the Frontal Assessment Battery test (FAB). In addition, the 36-item Short Form Health Survey (SF36) was completed by all patients enrolled and the physical and executive subset was used for correlation analysis. The 24 age-matched healthy controls (mean age 61.6 ± 9.2, range 36.6 – 71.9) had no history of a disease of the central nervous system, their neurological examinations were normal, and MRI T1 and T2 images revealed no pathological findings. Finally, clinical diagnosis of ALS was made according to the revised El-Escorial criteria. Disease characteristics of patients enrolled in the study are summarized in Table 1[10]. All subjects gave written informed consent to participate in the study, which was approved by the local ethics committee.

**Table 1:** Characteristics of ALS patients

**#**	**Age**	**Sex**	**Onset**	**El escorial**	**Disease duration mth**	**MMST**		**FAB**		**ALSFRS-R**		**SF36 physic**	
						**0**	**6**	**0**	**6**	**0**	**6**	**0**	**6**
1*	67.9	f	b	p	3	30		17		45	45	100	100
2	72.5	f	b	p	6	30	30	17	18	44		100	
3*	58.1	f	b	p	4	29	28	16	18	46	43	25	20
4*	72.0	m	l	p	31	30	29	17	17	39	32	35	35
5	75,9	m	l	p	15	29		12		25		5	
6*	64,8	m	l	d	33	29	28	15	12	32	27	0	0
7	64,4	m	l	p	32	30		16		43		20	
8*	32,7	m	l	d	19	30	30	18	18	30	27	0	0
9*	44,3	f	l	d	32	29	no	18	no	18	18	0	0
10*	64,5	f	l	s	3	28	27	15	9	45	35	10	5
11*	69,3	m	b	d	16	30	29	14	16	33	32	0	0
12*	70	f	l	s	37	27	30	15	18	35	31	10	10
13	68,6	f	b	p	7	21		12		47		100	
14*	67,5	m	l	p	22	29	28	18	18	45	45	95	95
15*	49,7	m	l	s	10	30		17		47		60	0
16*	64,8	f	l	d	52	30	30	18	18	37	37	0	0
17	48,3	f	l	d	104	no	no	no	no	21	21	0	
18*	71,3	m	b	p	8	27		15		40		35	15
19	60,2	m	l	p	18	29	30	15	18	39	39	25	
20	54,9	d	l	p	13	29		18		27		0	
21*	55,1	m	b	p	2	29		16		45		85	0
22*	64,1	f	b	d	13	30	30	15	15	34	25	45	30
23	74,4	m	l	p	107	30	30	17	17	32	32	30	
24*	65,3	f	b	p	28	29		18		21		0	15
**MW**	**61,5**				**25,6**	**28,9**	**29,2**	**16,0**	**16,3**	**36,3**	**32,6**	**32,5**	**20,3**
SD	10,9				27,8	1,9	1,1	1,8	2,8	9,0	8,3	37,2	32,2

S.D. standard deviation, f/m female/male ratio, s suspected, p probable, d definite, l limb onset, b bulbar onset, T0 (month 0), 6 follow-up (month 6), * patients, who underwent follow-up.

### Data acquisition

Images were acquired on a neuro-optimized 1.5-T GE Signa Horizon LX (General Electric Company, Milwaukee, WI, USA) using a 3-dimensional T1-weighted spoiled gradient recalled echo (SPGR) sequence generating 124 contiguous sagittal slices (RT 24ms; TE 8ms; flip angle 30°, 2 averages, acquisition time 13'10", in plane resolution 0.97 × 0.97 × 1.5 mm [3]). DTI was performed using echoplanar imaging (EPI) (39 contiguous slices, 3 mm thickness, 2 × 2 mm in plane resolution, 24 directions, b=1000, total scanning time 25 min). During scanning, all participants were placed comfortably and their heads were fixated within the headcoil using special cushions. All subjects received additional T2-weighted images to exclude cerebrovascular disease.

### Pre-processing

Data were processed on a standard IBM-compatible PC using SPM2 statistical parametric mapping software (Welcome Department of Cognitive Neurology, London) in the analysis environment MATLAB (version 6.1; the Math Works Inc, Natick, Mass). Images were reoriented into oblique axial slices aligned parallel to the anterior-posterior commissural axis with the origin set to the anterior commissure.

After calculatingthe FA and ADC maps, images were pre-processed and analyzed by statistical parametric mapping (SPM2) using an approach adopted from voxel-based morphometry as described herein by our group [11]. This included an optimized normalisation procedure, together with an automated exclusion of skull and CSF signal values and smoothing (6-mm FWHM). All EPI scans were normalized to the EPI-template provided by SPM and further used to create a site-specific EPI template appropriate to the population sample and with scanner specific image contrast. This site-specifc template was used again for normalisation and brain extraction for the individual images in the group studied, which resulted in optimal normalization and cleaning parameters for use with the FA and ADC images.

### Statistical analysis

Processed images of each tissue class were analyzed in the framework of the general linear model. ANCOVA was applied to compare the groups of patients and healthy controls (p < 0.05, corrected for the entire volume). Additionally, regression analyses with clinical measures were explored for ALS patients using the SPM2 model 'single subjects: covariates only'. In accordance with our anatomical a priori hypothesis regarding white matter changes in ALS along the CST, the statistical threshold was set at p < 0.001, corrected. For regions where changes could be expected in the follow-up (CST) [11,12], a small volume correction (SVC) was undertaken and the family-wise-error (FWE)-method (P < 0,05) for multiple comparisons was used.

## Results

### Clinical characterization

A total of 24 patients with sporadic ALS and the same number of age- and gender-matched healthy controls underwent MRI (T0) . In addition, DTI was performed in 15 patients with ALS after six months (follow-up). Based on MMST and FAB, no significant cognitive or executive impairment was observed in the T0 (MMST 28.8 ± 1.57, FAB 16.04 ± 1.80) and in the follow-up group (MMST 29.2 ± 1.07, FAB 16.31 ± 2.81). Three patients switched from possible to probable laboratory-supported ALS according to the revised El Escorial criteria. In the follow-up group, theALSFRS-R and the physical functioning SF36 subscore decreased from 35.4 (± 8.4) to 32,6 (± 8.3) and from 25,0 (± 33,0) to 20,7 (± 33,3) points, respectively after 6 months. Both reductions were not significant. Clinical characteristics of patients are summarized in Table 1.

### Whole-brain group differences in FA and ADC at T0

In the motor system, the FA in the ALS patient group (n=24) was reduced within the precentral gyrus, in the supplementary motor cortex, as well as in the CST at the level of corona radiata, pons, and crus cerebri compared to healthy controls. Further, FA was reduced in the corpus callosum, the parahippocampal gyrus and in frontal areas (Table 2).

**Table 2:** Results of group comparisons of ALS patients versus controls

	**Side**	**Cluster size**	**x**	**y**	**z**	**T**	**p**
**T0 FA all ALS patients (n=24) < controls**							
Corpus Callosum	l	7954	-10	-14	29	6.03	0.002
Corpus Callosum	l		-1	-16	25	5.35	0.003
Corpus Callosum	r		11	-16	29	5.29	0.003
Brainstem, Pons, CST	l	1589	-8	-27	-34	5.82	0.002
Brainstem, Pons, CST	r		9	-26	-36	4.54	0.007
Brainstem, Pons, CST	r		10	-22	-24	3.88	0.027
Precentral Gyrus, WM	r	2386	14	-21	58	5.69	0.002
subgyral, WM, Corona Radiata	r		27	-38	50	4.02	0.021
Medial Frontal Gyrus, WM, below BA 6	l	743	-12	-13	70	5.03	0.003
Precentral Gyrus, WM	r	290	48	4	8	4.59	0.007
Corona Radiata, Medial Frontal Gyrus, below BA 6	l	687	-35	-12	48	4.37	0.01
Precentral Gyrus, WM	l		-39	-19	48	4.18	0.015
subgyral, WM, below Gyrus postcentralis	l	201	-10	-37	61	4.35	0.011
Precentral Gyrus, subgyral, WM, Corona Radiata	l	343	-42	-15	23	4.34	0.011
Precentral Gyrus, WM	l	942	-28	-24	55	4.26	0.013
Frontal Lobe, subgyral, WM, Corona radiata	l		-17	-24	55	3.62	0.042
Frontal Lobe, subgyral, WM, Corona radiata	r	345	33	-8	38	3.87	0.028
Frontal Lobe, Precentral Gyrus, WM, Corona radiata	l	309	-39	-6	35	3.86	0.028
Frontal Lobe, Precentral Gyrus, BA 6	l		-46	1	40	3.82	0.03
Limbic Lobe, Parahippocampal Gyrus, WM	l	218	-20	-48	-11	3.80	0.032
**T0 ADC all ALS patients (n =24) > controls**							
Precentral Gyrus, WM	l	5108	-9	-25	67	5.99	0.02
Precentral Gyrus, subgyral, WM, below BA 6	l		-28	-16	65	5.5	0.02
Frontal Lobe, subgyral, WM, Corona radiata	l		-28	-25	50	5.33	0.02
Precentral Gyrus, subgyral, WM	r	5537	21	-16	65	5.56	0.02
Precentral Gyrus, WM	r		11	-23	67	5.22	0.02
Frontal Lobe, subgyral, WM, Postcentral Gyrus	r		15	-34	61	4.31	0.031
Brainstem, Pons	r	3243	6	-22	-28	5.13	0.02
Brainstem, Pons	l		-4	-23	-28	4.21	0.034
Limbic Lobe, Parahippocampal Gyrus, WM	r		26	-14	-14	3.98	0.041
Temporal Lobe, Superior Temporal Gyrus, GM	l	547	-62	-12	-1	4.90	0.02
Parietal Lobe, Superior Parietal Lobule, subgyral, BA 7	r	754	21	-50	65	4.73	0.022
Precentral Gyrus, WM	r	336	47	-2	31	4.55	0.025
Postcentral Gyrus, subgyral, WM	l	645	-18	-32	71	4.22	0.034
Postcentral Gyrus, subgyral, WM	l		-27	-31	67	4.02	0.04
Frontal Lobe, Superior Frontal Gyrus, GM, BA 9	l	467	-22	42	39	4.15	0.036
Frontal Lobe, subgyral, WM	l		-25	39	29	3.78	0.046
Temporal Lobe, subgyral, WM	l	409	-32	9	-35	4.11	0.037
Frontal Lobe, Superior Frontal Gyrus, WM	r	269	5	20	53	4.02	0.04
Frontal Lobe, Medial Frontal Gyrus, GM, BA 8	l	383	-6	27	53	4.02	0.04
Limbic Lobe, Uncus, WM	r	827	22	-3	-36	3.95	0.041
Limbic Lobe	r		22	-8	-43	3.68	0.049
Frontal Lobe, Precentral Gyrus, subgyral, WM	l	231	-51	-3	23	3.91	0.042
Sub-lobar, Insula, WM	l	208	-40	-17	19	3.89	0.043
Frontal Lobe, Superior Frontal Gyrus, GM	l	233	-22	10	57	3.88	0.043
Frontal Lobe, Middle Frontal Gyrus, GM, BA 8	l	217	-23	20	50	3.87	0.043
Frontal Lobe, Middle Frontal Gyrus, GM, BA 10	r	365	27	53	26	3.81	0.045
Frontal Lobe, Superior Frontal Gyrus, GM, BA 9	r		24	45	39	3.71	0.048
**Follow-up FA all ALS patients (n =15) < controls**							
Precentral Gyrus, subgyral, WM, Corona radiata	r	293	48	4	6	6.26	0.017
Corpus Callosum	l	3260	-8	-18	28	5.70	0.017
Corpus Callosum	r		11	-17	28	4.8	0.026
Left Brainstem, Pons	l	218	-6	-28	-38	5.02	0.02
Limbic Lobe, Parahippocampal Gyrus, WM	l	1004	-22	-42	-10	4.89	0.023
Temporal Lobe, Fusiform Gyrus, GM, BA 37	l		-27	-37	-16	4.26	0.04
Sub-lobar, Thalamus, GM, Pulvinar	l	217	-13	-33	1	4.51	0.033
Frontal Lobe, Medial Frontal Gyrus, WM, Corona radiata	r	696	13	-26	58	4.44	0.035
Frontal Lobe, Medial Frontal Gyrus, WM, Corona radiata	r		9	-27	69	4.28	0.04
parahippocampal Amygdala	r	206	29	-30	-26	4.36	0.037
Limbic Lobe, Parahippocampal Gyrus, GM, BA 34	r	204	17	-7	-19	4.20	0.043
**Follow-up ADC all ALS patients (n =15) > controls**							
Precentral Gyrus, WM	r	3258	24	-20	63	6.18	0.036
Precentral Gyrus, subgyral, WM	r		12	-21	62	5.01	0.001*
Precentral Gyrus, subgyral, WM, Corona radiata	r		18	-20	43	4.1	0.013*
Sub-lobar, Extra-Nuclear, WM, Internal capsule, Crus post.	r	379	18	-13	-1	4.32	0.008*
Sub-lobar, Extra-Nuclear, WM, Internal capsule, Crus post.	l	1234	-18	-13	-2	4.28	0.009*
Sub-lobar, Extra-Nuclear, WM, Internal capsule, Crus post.	l		-19	-12	6	4.13	0.012*
Precentral Gyrus, WM	l	351	-9	-25	65	4.27	0.009*
Precentral Gyrus, subgyral, WM, Corona radiata	l	1148	-37	-11	43	4.16	0.011*
Precentral Gyrus, subgyral, WM, Corona radiata	l		-28	-25	50	4.16	0.012*
Precentral Gyrus, subgyral, WM, Corona radiata	l		-25	-22	42	4.06	0.012*
subgyral, WM, Corona radiata, below BA 6	l	241	-13	-14	59	4.08	0.014*

Ordered by descending T-values. R=right; L=left, * SVC 10mm (FWE).

Diffusivity was enhanced in the motor system (precentral gyrus, premotor area, CST at the level of corona radiate, pons), the postcentral gyrus, and in frontal and temporal areas (Table 2). Enhanced diffusivity in the pons was rostrally located to pontine FA decrease.

### Whole-brain group differences in FA and ADC at follow-up

**Figure 1:**
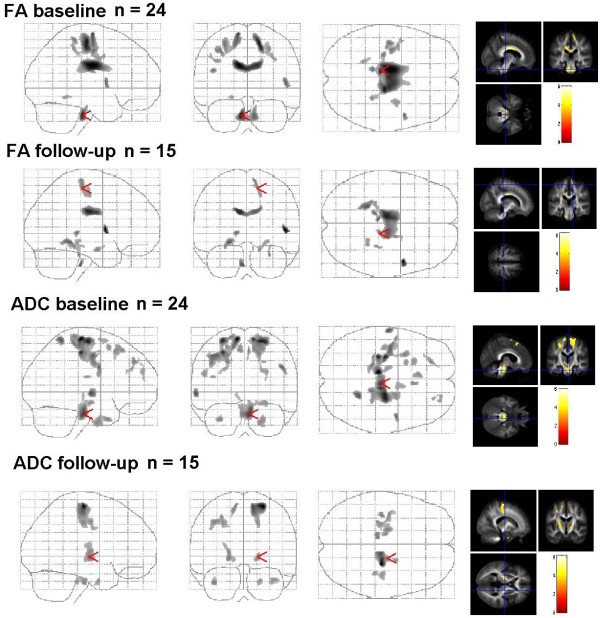
**Group comparisons of ALS patients versus controls.** A results are superimposed on the mean of all subjects of spatially normalized anisotropy images. Voxels with a significant decrease in FA in the patients versus controls are found in the precentral gyrus, the CST and pons (p < 0,001 uncorrected). ADC results are superimposed on the mean of all subjects of spatially normalized anisotropy images. Voxels with a significant increase in ADC in the patients versus controls are found in the precentral gyrus, CST, pons, frontal ares and limbic system (p < 0,001 uncorrected).

According to T0 MRI in ALS patients (n=15) after 6 months, the FA was reduced in the CST (corona radiate), the pons, the corpus callosum, and the parahippocampal gyrus. Additionally , FA was found decreased in frontal areas (gyrus frontalis medialis), insula, in the limbic system (amygdale), and in the thalamus at this time point (Table 2, Figure 1).

In the follow-up group, the diffusivity was again enhanced in the motor system (right precentral gyrus, and in the CST at the level of corona radiate, internal capsula). After SVC, there was a decrease in FA bilaterally in the WM of precentral gyrus, along the CST (corona radiate), and in the limbic system (fusiform gyrus, uncus) (Table 2, Figure 1). After 6 months, microstructural changes had progressed along the descending fibres and into the internal capsule.

### Comparison of T0 to follow-up

**Figure 2:**
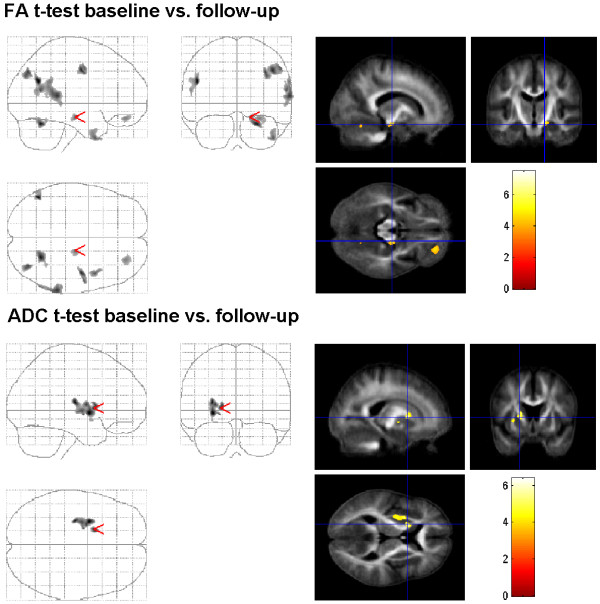
**Direct comparison between T0 and follow-up in ALS patients.** Comparison of FA results between T0 and follow-up (n=15) with paired t-test superimposed on the mean of all subjects of spatially normalized anisotropy images. FA is significantly reduced in the follow-up in the precentral gyrus, CST (at the mesencephal level), in the cerebellum (culmen, declive), the temporal and parietal lobe (p < 0,001 uncorrected). Comparison of ADC results between T0 and follow-up (n=15) with paired t-test superimposed on the mean of all subjects of spatially normalized anisotropy images. Diffusivity is significantly enhanced after 6 months in the internal and external capsule (p < 0,001 uncorrected).

Results of the 15 ALS patients, who had undergone follow-up DTI analysis were compared to their T0 data by means of the paired t-test. FA was significantly reduced at follow-up in the precentral gyrus, CST (at the mesencephal level), in the cerebellum (culmen, declive), the temporal and parietal lobe. Diffusivity was significantly enhanced after 6 months both in the internal and external capsule (Table 3, Figure 2).

**Table 3:** Results of direct comparisons of ALS patients and correlation analysis

	**Side**	**Cluster size**	**x**	**y**	**z**	**T**	**p**
**Paired t-test FA T0 / Follow-up**							
Temporal Lobe, Superior Temporal Gyrus, GM, BA 39	l	879	-49	-62	28	7.5	0.001*
Temporal Lobe, Superior Temporal Gyrus, GM, BA 39	l		-56	-64	16	4.82	0.033*
Parietal Lobe, Supramarginal Gyrus, GM, BA 40	l		-58	-61	30	4.19	0.075*
Cerebellum, Posterior Lobe, Declive	r	921	24	-59	-25	6.88	0.002*
Cerebellum, Anterior Lobe, Culmen	r		34	-52	-21	4.25	0.07*
Precentral Gyrus, WM	r	743	44	-9	41	6.67	0.003*
Precentral Gyrus, WM	r		53	-6	43	5.56	0.012*
Superior Temporal Gyrus, WM	r	2014	65	-39	10	6.36	0.004*
Superior Temporal Gyrus, GM, BA 22	r		61	-59	16	5.95	0.008*
Superior Temporal Gyrus, GM, BA 22	r		65	-48	20	5.12	0.022*
Parietal Lobe, Precuneus, WM	r	582	35	-74	39	6.21	0.005*
Brainstem, Midbrain, CST	r	270	16	-16	-17	5.89	0.008*
Temporal Lobe	r	756	44	8	-42	5.72	0.01*
Superior Frontal Gyrus, WM	r	221	35	39	35	5.27	0.018*
Middle Frontal Gyrus, subgyral, WM	r		29	34	31	4.76	0.035*
Superior Frontal Gyrus, GM, BA 11	r	487	28	45	-18	4.90	0.03*
Temporal Lobe, Middle Temporal Gyrus, WM	r	253	60	-7	-9	4.77	0.035*
Temporal Lobe, Superior Temporal Gyrus, WM	r		57	-2	3	4.33	0.063*
**Paired t-test ADC T0 / Follow-up**							
Extra-Nuclear, WM, External capsule	l	2054	-28	2	-3	6.38	0.003*
Extra-Nuclear, WM, External capsule	l		-29	-15	11	6.16	0.004*
Extra-Nuclear, WM, External capsule	l		-25	-1	8	5.5	0.01*
Extra-Nuclear, WM, Internal capsule	l	383	-19	8	3	5.94	0.005*

Ordered by descending T-values. R=right; L=left, * SVC 10mm (FWE).

### Correlation with clinical scores at T0

Disease duration negatively correlated with FA in precentral gyrus, CST (corona radiate, internal capsule) and the cerebellum, which means that in these areas FA decreases with time course of ALS. Changes in diffusivity did not correlate with disease duration. The ALSFRS-R positively correlated with FA in the CST, and the cerebellum. The ALSFRS-R did not correlate with diffusivity. Physical and executive function, reflected in SF36 subscore, positively correlated with FA in the cerebellum, but did not correlate with changes in diffusivity. This means that worsening ALS, indicated by a decrease of ALSFRS-R or SF36 subscore, is reflected as FA decrease in the CST and cerebellum (Table 4).

**Table 4:** Correlations with clinical data

	**Side**	**Cluster size**	**x**	**y**	**z**	**T**	**p**
**T0 FA correlation with ALSFRS-R**							
Right Brainstem, Midbrain	r	233	10	-24	-21	4.41	0.019*
Cerebellum, Posterior Lobe, Declive	r	251	13	-58	-19	3.99	0.04*
**T0 FA correlation with disease duration**							
Precentral Gyrus, WM, below BA 6	r	596	4	-27	70	4.93	0.007*
Precentral Gyrus, subgyral, WM, Corona radiata	r		6	-30	61	4.52	0.014*
Sub-lobar, WM, CST, Internal capsule	l	867	-31	-26	17	4.58	0.013*
Sub-lobar, WM, CST, Internal capsule	l		-30	-18	15	4.47	0.017*
Cerebellum, Anterior Lobe, Culmen	r	317	12	-56	-15	4.56	0.014*
Sub-lobar, WM, CST, Corona radiata	l	202	-13	-29	51	4.37	0.02*
**T0 FA correlation with SF36**							
Cerebellum, Anterior Lobe, Culmen	r	715	19	-43	-27	5.24	0.004*
Cerebellum, Anterior Lobe	r		10	-42	-33	4.4	0.019*
Cerebellum, Anterior Lobe	l	561	-10	-41	-33	4.84	0.008*
Cerebellum, Anterior Lobe, Culmen	l		-16	-42	-27	4.22	0.027*
**Follow-up FA correlation with ALSFRS-R**							
Precentral Gyrus, subgyral, WM, BA 6	r	469	50	2	38	6.35	0.006*
Precentral Gyrus, subgyral, WM, BA 6	r		46	-5	46	6.11	0.008*
Frontal Lobe, Middle Frontal Gyrus, WM	r	233	33	13	50	5.99	0.01*
Precentral Gyrus, WM, Corona radiata	r	417	29	-15	56	5.87	0.011*
Precentral Gyrus, WM, Corona radiata	l	219	-16	-12	59	5.01	0.014*
**Follow-up FA correlation with disease duration**							
Capsula Inerna, Crus post., CST	l	1722	-24	-19	2	6.87	0.003*
Sub-lobar, WM, Capsula Inerna, Crus post., CST	l		-28	-25	14	5.7	
Internal capsule, Crus post., CST	r	408	24	-16	-4	5.69	0.014*
Internal capsule, Crus post., CST	r		32	-17	-5	4.12	
External capsule	r	431	31	-12	10	5.61	0.015*
**Follow-up FA correlation with SF36**							
Brainstem, Pons, CST	l	387	-11	-32	-34	5.37	0.02*

Ordered by descending T-values. R=right; L=left, * SVC 10mm (FWE).

### Correlation with clinical scores at follow-up

After 6 months, the DTI data were correlated with clinical parameters. With respect to SVC, the FA negatively correlated with disease duration along the CST in the internal and external capsule. However, the decrease of FA was higher on the left side. Again diffusivity changes did not correlate with disease duration. ALSFRS-R positively correlated with FA in the precentral gyrus, the CST (corona radiate), the WM of right middle frontal gyrus and supplementary motor cortex. ALSFRS-R did not correlate with diffusivity changes. Physical and executive function (SF36 subscore) positively correlated with FA in the CST at brain stem level and negatively with diffusivity in the cerebellum (anterior lobe, dentatus, fastigium) and parahippocampal gyrus (Table 4).

## Discussion

Involvement of the white matter in ALS is increasingly being recognized and extensive white matter abnormalities can be found in the region of the central sulcus and the CST, extending across the corpus callosum and into the frontal lobes [1]. The intact axonal membrane and the myelin coating of axons are important determinants for anisotropic diffusion in neural tissues [13] and therefore, a reduced FA along the CST in ALS patients reflects axonal fibre degeneration and myelin pallor [1].

In the present study, microstructural changes in the brains of ALS patients could be demonstrated as decreased FA and increased diffusivity mainly in the pyramidal motor system, frontotemporal areas, limbic system, and the cerebellum. Comparison of the follow-up group with healthy probands revealed a progressive decrease in FA and diffusivity increase along the CST after 6 months. Increasing fiber tract pathology during disease course could be verified by direct comparison of T0 with the follow-up DTI data from 15 patients. Brain structure pathology correlated well with disease duration, ALSFRS-R andthe SF-36 subscore for physical and executive function, predominantly in the range of motor system, but interestingly also in the cerebellum and the limbic system.

### Widespread DTI changes in motoric and extramotoric systems in ALS

The decreased FA in the internal capsule and premotor cortex is in accordance with several DTI studies in ALS [4,6,8,9,14-23]. Microstructural alterations in frontal areas are in line with bilateral frontal atrophy in VBM [11,24-26], an increase of diffusivity in frontal regions [21,27] and observed in post mortem brain tissues [1]. Since the corpus callosum connects orbitofrontal and frontal cortices, its involvement in ALS is a consistent feature [9,28] and together with the known DTI changes in the hippocampal formations, cingulum, and frontal white matter, may reflect cognitive impairment, which is often recognized in patients with ALS [29,30]. However, aside from ruling out clinical significant dementia by means of MMSE and FAB, we did not undertake detailed neuropsychological testing.

### Involvement of cerebellum in ALS

The cerebellum has been little studied in ALS, probably because of the lack of cerebellar signs in most patients. However, evidence for the involvement of cerebellum in ALS derives from early pathological [31,32] and functional magnetic resonance imaging (fMRI) studies. Besides the enhanced activity in sensorimotor network, fMRI studies in ALS have demonstrated the recruitment of additional areas, such as the cerebellum and basal ganglia [33-37]. Hence, it is probable that functional compensation in ALS relies on existing resources and mechanisms and not on development of new synapses or pathways [35]. Structural changes of cerebellum in ALS could be demonstrated as regional white matter alterations [26] and decreased grey matter volume [38] in VBM and as increased diffusivity in the anterior lobe of the cerebellum bilateral [21].

In the present study, the direct comparison after 6 months showed a reduced FA in the cerebellum (culmen, declive) of ALS patients. These structural changes correlated with disease duration, ALSFRS-R, physical and executive functions. Since, the cerebellum contributes to coordination and improvement of motor performance and is involved in motor learning [39], the abnormal connectivity found between the supplementary motor cortex and cerebellar areas is therefore not only of functional, but also of a structural nature [35,37]. One could hypothesise that as a consequence of limited resources in the motor cortex, these cerebellar regions are activated due to an enhanced modulatory response in ALS. However, this compensation is paralleled by a progressive structural alteration of connectivity, finally leading to an ineffective modulation. However, the exact clinical and pathological relevance of these findings is not quite clear. We speculate that the structural changes in the cerebellum observed in the current study reflect a compensatory mechanism for the progressive loss of motor function. Further studies should evaluate cerebellar signs in more detail and undertake a comparison of cerebellar involvment in familal and sporadic ALS. Nevertheless with regard to studying cerebellar structures using DTI, several methodological limitations have to be taken into account such as the low resolution after normalization and the incomplete mapping of cerebellum. Moreover, measurement of radial and axial diffusivities would provide more specific information relating to diffusion tensor.

### Follow-up

The few existing longitudinal DTI studies have captured the progression of upper motor neurone degeneration in ALS by demonstrating decreasing FA over time along the CST [40-42]. Fibertracking of the mean CST showed a reduction of FA in a follow-up examination of ALS patients, indicating that degeneration of the CST appears to worsen over time [7]. In another six-month follow-up study, ALS patients showed DTI abnormalities that extended mainly into the bilateral frontal lobes [43]. Accordingly in our study, the disease progression was reflected in a progressive FA decrease along the CST, which correlated with disease duration and the decrease of ALSFRS-R. However, it is important to note that ALSFRS-R is a composite score of upper and lower motor neuron components, whereas FA changes along the CST may reflect more upon upper motor neuron degeneration. For further studies, it would be useful to correlate FA changes with ALSFRS-R subscores.

Furthermore, it remains unclear as to why regions with FA decrease (corona radiate, corpus callosum, brain stem) after 6 months appear smaller. In addition to method-based explanations, a possible interaction with swollen neighbouring motor neurones or astrocytes is also a plausible reason that could be discussed. Finally, we detected at 6 months follow-up that increase in diffusivity progressed in a caudal (CST) and frontal direction (premotor cortex). The direct comparison of follow-up patients with T0 data supports the observed progression of FA changes and revealed microstructural changes in regions which were not detected when comparing ALS patients with normal controls in the group comparison.

## Conclusions

In summary, our study provides further evidence for the well known structural changes in the CST and in frontal areas in the brains of ALS. Further, we demonstrate the involvement of cerebellum as a feature of disturbed connectivity in ALS, supported by the correlation analysis with clinical datasets. It seems that the extent of ALS-related DTI abnormalities is greater than those identified by VBM analysis [9,21], and that DTI can serve as stable biomarker in ALS [44].

## Abbreviations

ADC: Apparent diffusion coefficient; ALS: Amyotrophic lateral sclerosis; CST: Corticospinal tract; DTI: Diffusion tensor imaging; FA: Fractional anisotropy.

## Competing interests

Carsten Keil reports no disclosures. Tino Prell reports no disclosures. Thomas Peschel reports no disclosures. Viktor Hartung reports no disclosures. Reinhard Dengler reports no disclosures with regard to the current study. RD received honoraria, research grants, and travel grants from PharmAllergan, Ipsen Pharma, Merz Pharma, Boehringer-Ingelheim and Bayer Health Care. Julian Grosskreutz reports no disclosures.

## Authors’ contributions

Conceived and designed the experiments: JG, TP, CK, RD. Acquisition of data: CK. Analyzed the data: CK, JG, ThP. Wrote the paper: TP. All authors read and approved the final manuscript.
